# Validation of the Occupational Self-Efficacy Scale in a Sample of Chinese Employees

**DOI:** 10.3389/fpsyg.2021.755134

**Published:** 2021-11-01

**Authors:** Jiaxi Peng, Jiaxi Zhang, Xinzhou Zhou, Zhengwei Wan, Weizhuo Yuan, Junxiao Gui, Xia Zhu

**Affiliations:** ^1^College of Teachers, Chengdu University, Chengdu, China; ^2^Department of Political Theory, Xi’an Research Institute of High-Technology, Xi’an, China; ^3^College of Psychology, Southwest University, Chongqing, China; ^4^International College of Digital Innovation, Chiang Mai University, Chiang Mai, Thailand; ^5^College of Overseas Education, Chengdu University, Chengdu, China; ^6^Department of Military Medical Psychology, Air Force Medical University, Xi’an, China

**Keywords:** occupational self-efficacy scale, self-efficacy, item analysis, reliability, validity

## Abstract

Occupational self-efficacy, which refers to the belief that one is competent to fulfill work-related tasks or activities, has attracted increasing attention in recent years. The six-item version of the Occupational Self-Efficacy Scale (OSS-6) is an excellent tool for evaluating occupational self-efficacy; however, there is currently no report of the reliability and validity of the OSS-6 among Chinese people. This study aimed to translate the OSS-6 into Chinese and evaluate its reliability and validity in a sample of Chinese employees. A total of 433 junior staff at several firms completed the Chinese version of the OSS-6, the General Self-Efficacy Scale, the Rosenberg Self-Esteem Scale, the Minnesota Job Satisfaction Questionnaire, the in-role performance scale, and the career calling scale. Four weeks later, 94 participants were recalled and were retested using the OSS-6. Factor analysis results supported the one-factor model of the OSS-6. Excellent internal consistency was obtained with the OSS-6. Additionally, the OSS-6 results were significantly correlated with general self-efficacy, self-esteem, job satisfaction, in-role performance, and career calling. Furthermore, occupational self-efficacy was found to partially mediate the effects of career calling on job satisfaction and in-role performance. The results of this study supported the cross-cultural consistency of the structure of the OSS-6 and showed that the Chinese version of the OSS-6 demonstrated excellent validity and reliability. Therefore, the Chinese version of the OSS-6 can be used as an assessment tool for evaluating occupational self-efficacy in future studies.

## Introduction

Self-efficacy is the belief, judgment, and self-perception that one can accomplish a task (Hsu et al., 2019; Marsh et al., 2019). Self-efficacy can be divided into general self-efficacy and domain-specific self-efficacy (Löve et al., 2012; Azizli et al., 2015). Bandura (1993) maintains that activities differ among domains and that different activities require different abilities and skills; therefore, the self-efficacy of an individual may differ depending on the task. Self-efficacy is always related to specific domains (Paunonen and Hong, 2010). Compared with general self-efficacy, domain-specific self-efficacy can better predict people’s cognitive abilities and behaviors in specific domains (Paunonen and Hong, 2010; Grether et al., 2018). Hence, studies of self-efficacy have focused on specific domains, such as social self-efficacy, learning self-efficacy, and teaching self-efficacy (Siwatu, 2007; Iskender and Akin, 2010; Zimmerman and Kulikowich, 2016; Morris et al., 2017). In particular, the occupational self-efficacy domain has been attracting increasing attention (Schyns, 2004; Guarnaccia et al., 2018; Van Hootegem et al., 2021).

### Concept of Occupational Self-Efficacy

Occupational self-efficacy refers to the belief that an individual is competent to fulfill work-related tasks or activities (Felfe and Schyns, 2006). Occupational self-efficacy is not a specific personality trait or work capacity; rather, it is the confidence or belief in occupational capability (Schyns, 2004). Hackett and Betz (1981) were the first to propose the concept of occupational self-efficacy to explain gender differences in occupation selection among college students. They believed that there were insufficient proportions of female college students in traditionally male occupations because of females’ low self-efficacy in these domains. Because occupational tasks and activities involve various aspects, the concrete contents of occupational self-efficacy explored by researchers also vary. Generally, the existing research on occupational self-efficacy involves two aspects. The first aspect is self-efficacy related to occupational contents, or an individual’s belief in accomplishing the contents associated with an occupation (e.g., the education needed by an occupation, the concrete occupational task). The second aspect is self-efficacy related to the occupational behavior process, or an individual’s belief in accomplishing relevant occupational behaviors (e.g., career decision-making, occupation*-*seeking) and in achieving behavioral targets (Reese and Miller, 2006; Çetin and Aşkun, 2018; Kim and Lee, 2018). In most studies, occupational self-efficacy has been measured by participants’ belief in their capabilities to complete what they consider to be the broad requirements of the work (Fletcher et al., 1992). Researchers have actualized the construct of occupational self-efficacy as a general entity, not in terms of specific tasks (Çetin and Aşkun, 2018).

### Measurement of Occupational Self-Efficacy

So far, the main tools used to evaluate occupational self-efficacy include the perceived employability scale (Rothwell et al., 2008), the occupational self-efficacy index (Fletcher et al., 1992), and the task-specific occupational self-efficacy scale (Osipow and Temple, 1996), which differ in applicable targets and evaluation methods. For instance, the perceived employability scale mainly measures the belief that an individual can successfully cope with different scenarios and perform behaviors that will promote their occupational development (Berntson and Marklund, 2007). This scale involves four dimensions, including interpersonal efficacy, information-gathering and barrier-removal efficacy, persistence, and goal-setting efficacy, and it is mainly applicable to groups of adults who earn low incomes (Daniels et al., 1998).

However, these evaluation tools have several problems, such as the large number of items, their unclear constructs, their lack of cross-cultural consistency, and the niche groups to which they can be applied (Richard et al., 2011). The original Occupational Self-Efficacy Scale (OSS), which was developed by Schyns and Collani (2002), consists of 20 items. The instrument proved to be good at measuring various characteristics of occupational self-efficacy (Schyns and Collani, 2002). Subsequently, short forms of the OSS, consisting of six or eight items, were developed (Rigotti et al., 2008). Various empirical studies have shown that the six-item version of the OSS (OSS-6) has equally good reliability and validity when compared to the original 20-item version (Schyns and Collani, 2002; Rigotti et al., 2008), and it has become the most widely used occupational self-efficacy evaluation tool.

The OSS-6 has some advantages over other existing scales for evaluating occupational self-efficacy. First, it is the smallest scale used to evaluate this construct and thus permits the inclusion of other variables in the same research without overloading study participants. Second, it is composed of a single dimension to assess occupational self-efficacy, which allows for its use in different occupational settings. This advantage is especially relevant given the wide variety of jobs in the contemporary world. Third, the OSS-6 has been developed for particular working contexts, especially organizations, so it has specific application value for studies of teamwork or customer relations. Rigotti et al. (2008) compared the use of the OSS-6 in five languages in five countries, including Germany, Sweden, the United Kingdom, Belgium, and Spain, and found high reliability and validity among the five versions, suggesting that the OSS has high cross-cultural consistency. Damásio et al. (2014) adapted and validated the Brazilian version of the OSS-6, which exhibited good reliability and validity for measuring occupational self-efficacy in Brazil.

### Aim of the Current Study

With regard to China, few scales are available to evaluate occupational self-efficacy (Rigotti et al., 2008). Although a few measurements have been validated for the Chinese context, they tend to evaluate self-efficacy for possible career decision-making (Zhang and Schwarzer, 1995; Li et al., 2014). Due to the well-known and important associations for the measurement of occupational self-efficacy described above, an increasing effort has been made toward the effective measurement of occupational self-efficacy. However, until now, few scales have been available to evaluate occupational self-efficacy, and no study has evaluated the reliability and the validity of the OSS among Chinese people. Since the OSS-6 is outstanding due to its small item volume, clear construct (single dimension), and high reliability and validity, this version was translated and revised as part of the present study. Its reliability and validity under the Chinese cultural background needed to be evaluated to see whether it could be an efficient tool to measure the perception of individuals’ abilities to effectively perform work tasks. Thus, the focus of the present study was to adapt and verify the validity the OSS-6 in the Chinese context.

Occupational self-efficacy is a special self-efficacy in the work context, and it involves people’s beliefs about their abilities to effectively perform their work tasks, which are highly correlated with their beliefs about their competencies to organize and execute the courses of behaviors required to produce given achievements (Bandura, 1977). Shelton (1990) maintains that self-efficacy interacts with other variables, such as self-esteem, job satisfaction, and in-role performance. With a higher self-evaluation or self-esteem, the individual is more confident in accomplishing tasks at work (Joseph et al., 2014). Hence, general self-efficacy and self-esteem could be treated as the major content validity criteria for occupational self-efficacy. In this study, we hypothesized that the measurement of occupational self-efficacy in Chinese culture would be significantly correlated with self-efficacy and self-esteem, which we regarded as the content validity criteria. At the same time, occupational self-efficacy can adjust cognition, motivation, and emotion processes and thereby affect occupational identity, work performance, job attitude, and work enthusiasm (Schyns, 2004; Hirschi, 2012). Occupational self-efficacy is significantly correlated with job satisfaction (Dendinger et al., 2005) and job performance (König et al., 2010; Park et al., 2016; Burić and Moe, 2020). Thus, the current study further hypothesized that the results of the measurement of occupational self-efficacy via the OSS-6 in Chinese workers would significantly correlate with job satisfaction and performance. Significant correlations between occupational self-efficacy and job satisfaction or in-role performance were selected as the criterion validity criteria for occupational self-efficacy measurement in Chinese culture.

Career calling is another variable that has a close relationship with occupational self-efficacy, and it has an important prepositional effect on occupational self-efficacy. Career calling is defined as “being called to do works that are morally and socially meaningful,” and it is viewed as a strong and meaningful kind of passion one experiences from working (Dobrow and Tosti-Kharas, 2011). The endorsement of a calling can enhance occupational self-efficacy since those with high career calling are more likely to be able to resolve unexpected obstacles because of their clear sense of purpose (Park et al., 2016). Further, career calling contributes to career development, job satisfaction, and in-role performance (König et al., 2010; Çetin and Aşkun, 2018; Peng et al., 2020). Therefore, occupational self-efficacy mediates the relationship between career calling and job satisfaction. Park et al. (2016) proved this assumption and found that occupational self-efficacy significantly mediates the effects of career calling on job performance and organizational citizenship behavior in salespeople. Thus, we also hypothesized that career calling and occupational self-efficacy would be significantly and positively correlated. Additionally, since occupational self-efficacy can mediate the effects of career calling on job satisfaction and in-role performance, we further hypothesized that there would be mediating effects in the relationships between career calling and job satisfaction or in-role performance, which were also regarded as the validity criteria for the Chinese version of the OSS-6.

## Materials and Methods

### Participants and Procedures

This field study used a cross-sectional design and convenience sampling. The study was conducted in different departments of firms in Chengdu, China, using a pencil and paper test. In the research group, 487 employees were selected by simple random sampling. The inclusion criteria were: (1) the participant worked as a full-time employee and (2) the participant volunteered to partake in this research. The enterprise domains included decoration, foods, environmental protection, and logistics transportation. In total, 487 copies of the scale were distributed, and 433 (88.91%) valid response copies were returned. Among the valid copies, there were 298 male responders and 135 female responders. The participants ranged in age from 24 to 46 years, with a mean age of 29.74 years (standard deviation = 8.49). There were 274 (63.27%) participants who had received a bachelor’s degree or above. The average length of employment was 37.43 months. All of the subjects were given 10 RBM (about 1.5 US dollars) for their participation. 94 participants were recalled and retested 4 weeks later. The participants together completed the Chinese version of the OSS-6 and the criterion validity assessments in meeting rooms.

The questionnaires were distributed by a research assistant and psychology students. Before finishing the questionnaires, the participants were briefly notified of the research purpose and methodology. All of the participants read and signed an informed consent document before participating in the study. All of the participants filled in the questionnaires anonymously. The research described in this paper meets the ethical guidelines of Chengdu University and has been approved by the university’s Ethics Committee (reference number: CDU20201821SJ).

### Measurements

#### The Chinese Version of the OSS-6

The original version of the OSS developed by Schyns and Collani (2002) consists of 20 items. Rigotti et al. (2008) revised and formulated a brief OSS with six items. The translation and revision process consisted of three steps. To form the first draft, three Ph.D. candidates majoring in psychology and one professor majoring in English independently translated the scale, and the three translation copies were compared and combined. To form the second draft, two psychology professors and two Chinese language professors corrected the accuracy and fluency of Chinese grammar and words in the first draft, making it more consistent with the phraseological rules of Chinese. To form the final version, two English-to-Chinese translators independently back-translated the second draft, and together with two psychology experts, they evaluated, discussed, and fine-tuned the draft until the revised draft was not different from the original version and the items were more understandable. Thus, the Chinese version of the OSS-6 was established. Some example items are “I meet the goals that I set for myself in my job” and “I feel prepared for most of the demands in my job.” Participants’ responses were rated using a six-point scale ranging from 1 (completely not true) to 6 (completely true). A total of 20 college students were recruited to evaluate the intelligibility of each item, and 100% of these participants considered the language of the Chinese version of the OSS-6 to be comprehensible. The overall Cronbach’s alpha coefficient in this study was 0.85.

#### General Self-Efficacy Scale

The General Self-Efficacy Scale (GSE) consists of 10 items that assess optimistic self-beliefs to cope with a variety of difficult demands in life with statements such as “I can usually handle whatever comes my way” (Schwarzer and Jerusalem, 1995; Weber et al., 2013). Participants’ responses were rated using a four-point scale ranging from 1 (does not describe me at all) to 4 (describes me to a great extent). In the present study, the Cronbach’s alpha coefficient for the GSE was 0.86.

#### Rosenberg Self-Esteem Scale

The Rosenberg Self-Esteem Scale (RSES) involves 10 items, five of which were scored reversely in the present study (Robins et al., 2016). Some examples of items include “On the whole I am satisfied with myself” and “All in all, I am inclined to feel that I am a failure (scored reversely).” The participants’ responses were rated using a four-point scale ranging from 1 (strongly disagree) to 4 (strongly agree). The RSES, which has been translated into Chinese with proven high reliability and validity, has been used extensively (Zhang et al., 2019). In this study, the Cronbach’s alpha coefficient for the RSES was 0.90.

#### Minnesota Satisfaction Questionnaire

The Minnesota Satisfaction Questionnaire, which consists of 20 items, was used to measure job satisfaction. Examples of items on this assessment include “The chance to try out some of my own ideas” and “The chances of advancement/promotion in this position.” Items were rated using a five-point scale ranging from 1 (strong dissatisfaction) to 5 (strong satisfaction). This widely used scale shows good validity and reliability (Peng et al., 2019b). In the current study, the Cronbach’s alpha coefficient for this scale was 0.87.

#### In-Role Performance Scale

The in-role performance scale, developed by Williams and Anderson, consists of six items (Williams and Anderson, 1991). Example items include “Adequately completes assigned duties” and “Meets formal performance requirements of the job.” Items were rated using a seven-point scale ranging from 1 (strongly disagree) to 7 (strongly agree). The in-role performance scale, which was translated into Chinese in a previous study, showed good validity and reliability (Xingyong et al., 2017). In the current study, the Cronbach’s alpha coefficient for this scale was 0.91.

#### Career Calling Scale

The career calling scale, developed by Dobrow and Tosti-Kharas (2011), consists of 12 items. Some example items include “The first thing I often think about when I describe myself to others is that I’m a decoration worker/deliveryman/environment protection worker” and “I enjoy doing my current work more than anything else.” Item responses ranged from 1 (strongly disagree) to 7 (strongly agree). The career calling scale, which was previously translated into Chinese, has shown good validity and reliability (Peng et al., 2020). In the current study, the Cronbach’s alpha coefficient for this scale was 0.85.

### Data Analysis

The descriptive analysis, correlation analysis, *t*-test, and exploratory factor analysis performed in this study were conducted using SPSS 18.0. The confirmatory factor analysis and path analysis were performed using AMOS17.0. The bootstrap test was used to assess mediating effects. *P* < 0.05 or *P* < 0.01 were considered to be statistically significant. According to Hu and Bentler (1999), a model was considered to have reasonably good fit if all the path coefficients were significant at the levels of *P* < 0.05, root mean square error of approximation (RMSEA) < 0.08, and confirmatory fit index (CFI) > 0.95.

## Results

### Test of Common Method Bias

The Harman’s single-factor test was used to assess for the presence of common method bias. All the items of all the scales used in this study were involved in the exploratory factor analysis, which showed that seven factors had an eigenvalue > 1 and that the variance explained by the first factor was 22.67%, which was below the critical value of 40%, suggesting that there was an insignificant level of common method bias.

### Item Analysis

The item analysis was based on the item score-total score correlation coefficients and the critical ratio values. The participants were divided into a high-score group (the top 27% ranked by total scores) and a low-score group (the bottom 27% ranked by total scores), and an independent *t*-test was conducted for each item’s scores between the high-score and low-score groups. The results showed that the correlation coefficients between each item and the total scores of the OSS-6 were 0.6*–*0.90. A critical ratio test showed that the item scores were all significantly different between the high-score and low-score groups (*P* < 0.01), suggesting that all items had high discriminability (Table 1). In addition, the factor loading of different items in the exploratory factor analysis showed significance at the level of *P* < 0.01 for all (Table 1).

**TABLE 1:** Correlation coefficients, critical ratio values and factor loading of items in exploratory factor analysis.

Item	Mean	*SD*	*r* with the total score (*n* = 433)	CR (*n* = 433)	Factor loading (*n* = 216)
1	4.31	1.17	0.84**	17.57**	0.87
2	3.79	1.28	0.86**	28.41**	0.88
3	2.72	1.27	0.66**	19.82**	0.70
4	4.44	1.15	0.78**	11.94**	0.80
5	3.93	1.08	0.65**	22.82**	0.60
6	4.24	0.91	0.71**	21.71**	0.69
Characteristic root					5.11
Variance explained					60.94%

**P < 0.01.

### Exploratory Factor Analysis

The data sample was divided randomly into part A and part B, which included 217 and 216 valid data, respectively. First, an exploratory factor analysis was performed on the data of part A. The KMO was 0.84, suggesting that this scale was suitable for factor analysis. Scree plots showed that only one factor had an eigenvalue > 1; the loads of all items under this factor exceeded 0.6 (*P* < 0.01) (Table 1), and this factor could explain 60.94% of the variance.

### Confirmatory Factor Analysis

**FIGURE 1:**
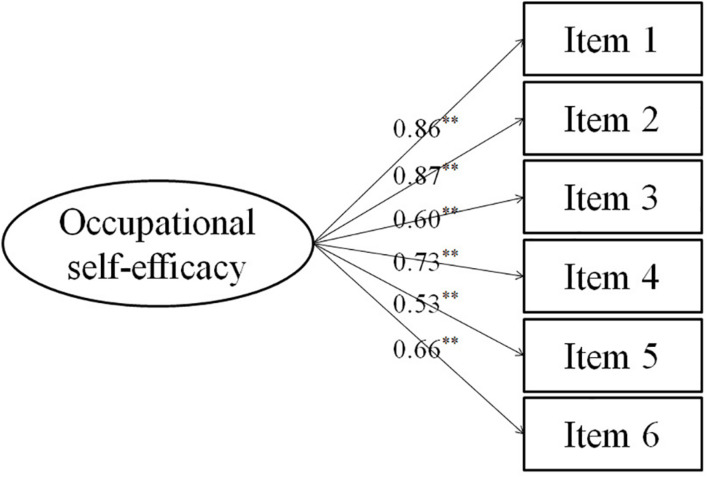
Confirmatory factor analysis. **P < 0.01. The factor loading was the value from confirmatory factor analysis.

A confirmatory factor analysis was performed on the data of part B to evaluate the fitness of the single-factor model (Figure 1). The results showed that all fitness indices demonstrated statistical significance, suggesting that the single-factor model fit the data well. The fitness indices of the Chinese version of the OSS-6 were compared with those of the German, English, Spanish, Swedish, and Belgian language versions (Table 2), and the model fit index results were almost the same as those of the other versions of the OSS-6.

**TABLE 2:** Fitness statistics for the confirmatory factor analyses of OSS-6.

	Chinese (*n* = 217)	Germany (*n* = 200)	Sweden (*n* = 147)	Belgium (*n* = 616)	Britain (*n* = 195)	Spain (*n* = 377)
χ^2^/df	4.53	4.77	2.94	14.09	5.36	3.43
GFI	0.95	0.93	0.94	0.93	0.93	0.97
AGFI	0.88	0.84	0.86	0.83	0.83	0.94
CFI	0.95	0.94	0.95	0.92	0.94	0.98
90% CI RMSEA	0.09 to 0.17	0.10 to 0.18	0.07 to 0.17	0.12 to 0.17	0.11 to 0.19	0.05 to 0.11
RMSEA *p* values	0.00	0.00	0.02	0.00	0.00	0.05

### Analysis of Reliability

An analysis of the internal consistency coefficient showed that the Cronbach’s α of the Chinese version of the OSS-6 was 0.85 and that the test-retest reliability after 4 weeks was 0.82.

### Analysis of Validity

The validity of the Chinese version of the OSS-6 was evaluated in terms of construct validity, convergence validity, and criterion validity. The validation factor analysis showed that the Chinese version of the OSS-6 had high construct validity. The convergence validity was characterized by the average variance extracted (AVE). Based on AVE = Σλ^2^/n (where n is the number of items and λ is the standardized factor load), the AVE of the OSS-6 was 0.58 (greater than the critical value of 0.5), suggesting that this scale had high convergence validity (Izogo, 2016). A correlation analysis demonstrated that occupational self-efficacy was intermediately positively correlated with general self-efficacy, self-esteem, job satisfaction, in-role performance, and career calling (*r* > 0.35, *P* < 0.01) (Table 3).

**TABLE 3:** Correlation analysis between occupational self-efficacy and criterions (*n* = 433).

	General self-efficacy	Self-esteem	Job satisfaction	In-role performance	Career calling
Occupational self-efficacy	0.52**	0.38**	0.55**	0.44**	0.43**

**P < 0.01.

**FIGURE 2:**
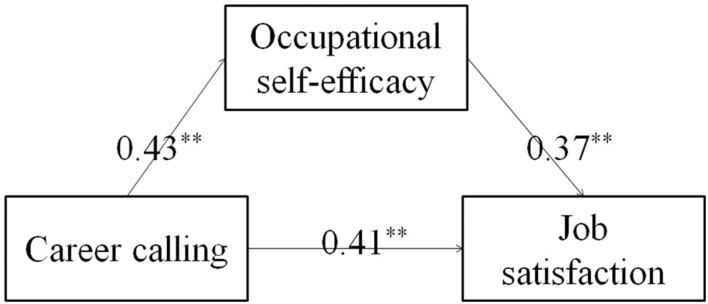
The mediating effect of occupational self-efficacy between career calling and job satisfaction. **P < 0.01.

**FIGURE 3:**
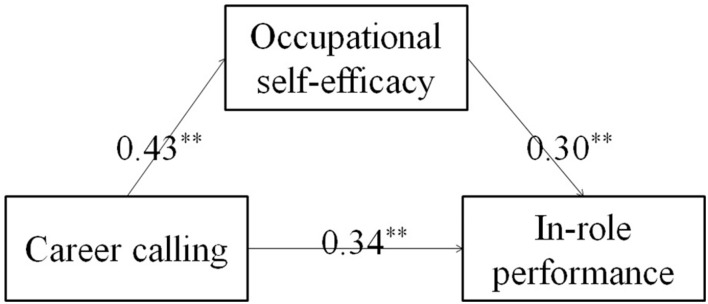
The mediating effect of occupational self-efficacy between career calling and in-role performance. **P < 0.01.

To explore the mediating role of occupational self-efficacy in the relationship between career calling and job satisfaction, and between career calling and in-role performance, the path analysis was conducted (Figures 2, 3). The results showed that all the path coefficients were significant at the level of *P* < 0.05. The bootstrap method was used to further estimate the mediating effect. The standardized direct and indirect effects of the mediating effect models are shown in Table 4. The bootstrap test results showed that the 95% confidence intervals for all the direct effects did not overlap with zero. The results also showed that the 95% confidence intervals for the indirect effect of career calling on the job satisfaction through occupational self-efficacy were 0.10–0.23, which again did not overlap with zero. The 95% confidence intervals for the indirect effect of career calling on the in-role performance through occupational self-efficacy were 0.08–0.18, which also did not overlap with zero. Together, these results showed that all direct and indirect effects were significant at the level of *P* < 0.05. According to Anderson and Gerbing (1988), it can be concluded that occupational self-efficacy partially mediated the effects of career calling on job satisfaction and in-role performance.

**TABLE 4:** Standardized effect and 95% CIs for the final model.

Model	Model pathways	Estimated effect	95% CI	
			Lower bonds	Up bonds
Job satisfaction as dependent variable	**Direct effect**			
	Career calling→ Occupational self-efficacy^a^	0.43	0.32	0.53
	Occupational self-efficacy→ Job satisfaction^a^	0.37	0.26	0.48
	Career calling→ Job satisfaction ^a^	0.41	0.31	0.52
	**Indirect effect**			
	Career calling→ Occupational self-efficacy →Job satisfaction^a^	0.16	0.10	0.23
In-role performance as dependent variable	**Direct effect**			
	Career calling→ Occupational self-efficacy^a^	0.43	0.32	0.53
	Occupational self-efficacy→ In-role performance^a^	0.30	0.20	0.40
	Career calling →In-role performance^a^	0.34	0.23	0.46
	**Indirect effect**			
	Career calling→ Occupational self-efficacy →In-role performance^a^	0.13	0.08	0.18

*^*a*^Empirical 95% confidence interval does not overlap with zero.*

The *t*-test results showed that the OSS-6 scores of participants with a bachelor’s degree or above were significantly higher than those of other participants (*t* = 6.18, *P* < 0.01, Cohen’s *d* = 0.60). The participants were ranked by length of employment (in work years), and the top 50% (9.32 ± 3.86 years) and the bottom 50% (3.15 ± 2.74 years) were considered senior employees and junior employees, respectively. The results showed that the scores of the OSS-6 were significantly different between groups (*t* = 5.75, *P* < 0.01, Cohen’s *d* = 0.55) and that senior employees demonstrated higher occupational self-efficacy than junior employees (Table 5).

**TABLE 5:** Group differences in OSS-6 scores ($\bar{x}$ ± SD).

	OSS-6 scores	*T*	Cohen’ *d*
Bachelor or above (*n* = 274)	24.55 ± 4.69	6.18**	0.60
Others (*n* = 159)	21.49 ± 5.42		
Senior group (*n* = 216)	24.81 ± 4.85	5.75**	0.55
Junior group (*n* = 217)	22.05 ± 5.14		

**P < 0.01.

## Discussion

In the current study, the OSS-6 was translated and revised. Item analysis, reliability tests, and validity tests were conducted. Self-efficacy is always associated with specific domains (Paunonen and Hong, 2010). Occupational self-efficacy, which is self-efficacy in the occupational domain, can predict work performance and job satisfaction well and is an important topic in work studies (Soeker, 2016; Tomas et al., 2019). The OSS-6 is an excellent tool for evaluating occupational self-efficacy and has high application value (Rigotti et al., 2008). This study offers an effective tool for performing relevant research on occupational self-efficacy in China, and it validates the applicability of the OSS-6 in Chinese culture, which further supports its cross-cultural consistency.

First, item analysis results showed that each item score was significantly and positively correlated with the total score. The high-score group and the low-score group were significantly different when it came to each item score. These results verified the high item quality of the Chinese-version of the OSS-6. The exploratory factor analysis results showed that the Chinese version of the OSS was one-dimensional and that the factor loads of all items were above 0.60. The principal component could explain 60.94% of the variance, suggesting that the item content was clear and highly interpretable (Peng et al., 2019a).

As for reliability, the Chinese version of the OSS-6 had a test–retest reliability of 0.82 and an internal consistency coefficient of 0.85, which were insignificantly different from the German version (α = 0.87) and English version (α = 0.90), suggesting that the Chinese version of the OSS had high stability and consistency (Rigotti et al., 2008). As for validity, the confirmatory factor analysis results showed that the single-factor model of the OSS-6 fit the data well, suggesting that the OSS-6 had high construct validity. The AVE was 0.58 (greater than the critical value of 0.50), suggesting that this scale converged well (Izogo, 2016). Theoretically, occupational self-efficacy is significantly and positively correlated with both general self-efficacy and self-esteem and can significantly affect employees’ job satisfaction, job performance, and career calling (Schyns and Collani, 2002; Dendinger et al., 2005; Agrawal et al., 2012). The general self-efficacy scale, self-esteem scale, and other scales were selected as the assessment criteria. The results showed that occupational self-efficacy was positively correlated with general self-efficacy, self-esteem, job satisfaction, in-role performance, and career calling, suggesting to some extent that the OSS-6 had high validity. Similar to previous studies, in the current study, path analysis and bootstrap test results revealed that occupational self-efficacy partially mediated the effects of career calling on job satisfaction and in-role performance (Domene, 2012; Ngo and Hui, 2018; Sari, 2019). Hall and Chandler (2005) proposed the career success model and suggested that employees who regarded their work as a calling were more competent at work because of their clear sense of purpose and focused task efforts. Since previous studies have documented that the endorsement of a calling enhances self-efficacy, and since both career calling and occupational self-efficacy significantly predict job satisfaction and job performance, it is logical to hypothesize that occupational self-efficacy can mediate the effects of career calling on job satisfaction and in-role performance (Skaalvik and Skaalvik, 2017; Xie et al., 2017; Burić and Moe, 2020). In the current study, we evaluated and verified the significant mediating effects using the OSS-6. The results shed some light on how career calling correlated with job satisfaction and in-role performance, and they also showed to some extent the high validity of the Chinese version of the OSS-6.

Previous studies have verified that self-efficacy originates from the experience of success, as well as from positive attribution style and self-assessment (Weiser and Riggio, 2010). Employees with high education levels generally have high self-evaluations and may believe that they have high competence (Kogut, 2016), and thus they have high occupational self-efficacy. Employees with longer employment (in work years) and richer work experience are more capable of handling difficulties in jobs than those with shorter employment and less rich work experience, and thus they have higher occupational self-efficacy (Dierdorff and Surface, 2007; Karin et al., 2018). The OSS-6 scores were shown to be significantly different among employees with different education levels and among employees with different work experiences, suggesting the high validity of the OSS-6.

## Conclusion

The purpose of the current study was to adapt and provide evidence of the validity of the Chinese version of the OSS-6. The results showed that all the items of the Chinese version of the OSS-6 had good discriminant ability, and the OSS-6 demonstrated adequacy in terms of reliability, construct validity, content validity, and criterion validity. The strengths of the current study include the robust methods of data analysis. Exploratory and confirmatory factor analyses, along with the bootstrap test, were performed to test the mediating effect of occupational self-efficacy between antecedent and dependent variables, and they reinforced the power and reliability of the presented results. The OSS-6 can be used as an assessment tool for evaluating occupational self-efficacy in China in future studies. The limited sample size was the main limitation of the present study. Future studies should inspect larger and more diverse samples to support or dispute the data presented in this study.

## Data Availability Statement

The raw data supporting the conclusions of this article will be made available by the authors, without undue reservation.

## Ethics Statement

The studies involving human participants were reviewed and approved by the research described in this paper meets the ethical guidelines of Chengdu University and has been approved by its Ethics Committee (reference number: CDU20201821SJ). The patients/participants provided their written informed consent to participate in this study.

## Author Contributions

JP, JG, and XZ conceived and designed the study, contributed reagents, materials, and analysis tools and wrote the manuscript. JP, XZZ, and ZW collected the data. JP, JZ, and WY revised the manuscript. All the authors contributed to the article and approved the submitted version.

## Conflict of Interest

The authors declare that the research was conducted in the absence of any commercial or financial relationships that could be construed as a potential conflict of interest.

## Publisher’s Note

All claims expressed in this article are solely those of the authors and do not necessarily represent those of their affiliated organizations, or those of the publisher, the editors and the reviewers. Any product that may be evaluated in this article, or claim that may be made by its manufacturer, is not guaranteed or endorsed by the publisher.
